# Extracellular MicroRNAs as Potential Biomarkers for Frail Kidney Phenotype: Progresses and Precautions

**DOI:** 10.14336/AD.2023.0818

**Published:** 2024-08-01

**Authors:** Chia-Ter Chao, Chih-Kang Chiang, Kuan-Yu Hung

**Affiliations:** ^1^Nephrology division, Department of Internal Medicine, National Taiwan University Hospital, Taipei, Taiwan.; ^2^Nephrology division, Department of Internal Medicine, National Taiwan University College of Medicine, Taipei, Taiwan.; ^3^Graduate Institute of Toxicology, National Taiwan University College of Medicine, Taipei, Taiwan.; ^4^Nephrology division, Department of Internal Medicine, National Taiwan University Hospital BeiHu branch, Taipei, Taiwan.; ^5^Center of Faculty Development, National Taiwan University College of Medicine, Taipei, Taiwan.; ^6^Blood purification division, Department of Integrative Diagnostics and Therapeutics, National Taiwan University Hospital, Taipei, Taiwan.; ^7^Nephrology division, Department of Internal Medicine, Shuang Ho Hospital, Taipei Medical University, Taipei, Taiwan.; ^8^Department of Internal Medicine, School of Medicine, Taipei Medical University, Taipei, Taiwan.

**Keywords:** biomarker, chronic kidney disease, end-stage kidney disease, frailty, geriatrics, miRNAs

## Abstract

Frailty describes the cumulative subtle health deficits leading to an increased vulnerability to insults among older individuals or disease-laden ones. The prevalence of frailty increases substantially and relentlessly over declining renal function. Frailty in patients with chronic kidney disease (CKD) carries kidney-specific risk factors, clinical correlates and outcomes associations, hence alternatively termed frail kidney phenotype by researchers. Pathogenetically, miRNAs participate extensively in the development and aggravation of frailty, including the occurrence of frail kidney phenotype in CKD patients. These understandings spark profound interest in discovering biomarkers for identifying this detrimental phenotype, and extracellular miRNAs emerge as potentially useful ones. Pilot studies identify promising miRNA candidates for evaluating intermediates and surrogates of frail kidney phenotype, and more are underway. Several potential miRNA species in biologic fluids, such as circulating miR-29b and miR-223 (as inflammatory markers), exosomal miR-16-5p, miR-17/92 cluster members, and miR-106-5p (for uremic vasculopathy), serum exosomal miR-203a-3p (for uremic sarcopenia) have been examined and can be promising choices. Nonetheless, there remains research gap in affirming the direct connections between specific miRNAs and frail kidney phenotype. This stems partially from multiple limitations less well acknowledged before. From this perspective, we further outline the limitations and precautions prior to validating specific extracellular miRNA(s) for this purpose, from the definition of frailty definition, the functional and tissue specificity of miRNAs, the severity of CKD, and various technical considerations. It is expected that more affirmative studies can be produced for extending the utility of extracellular miRNAs in predicting frail kidney phenotype.

## Frailty and frail kidney phenotype

Frailty denotes the degenerative process of cumulative subtle health deficits, leading to phenotypic abnormalities involving various combinations of musculoskeletal, nutritional, cognitive, and other domains [1]. Although the operationalization of frailty can be achieved through physical capacity ascertainment (termed “frail phenotype” suggested by Fried et al. [2]) or deficit counting measurement (termed “frailty index” [3]), there remains a wide research gap between how we identify frailty and how we should treat this detrimental syndrome. Frailty has been touted as the underlying susceptibility trait of and a mediator for other geriatric syndromes, falls, and disability [4,5]. Indeed, frailty among affected older adults confers a higher risk of functional deterioration, institutionalization, prolonged hospitalization, and long-term mortality compared to those without, and even the transitional state, pre-frailty, exhibits modest yet significant influences [6]. These adverse effects are also observed in patients with secondary frailty, that is, those who are not elderly but with morbidities such as diabetes mellitus (DM) [7]. In light of the high prevalence of pre-frailty and frailty among community-dwelling older adults (~20%) and those in other healthcare settings [8], calls to earlier identify frailty has assumed priority for primary care clinicians and have been endorsed by professional societies and health authorities [9]. Treatments and care strategies for physical frailty have become an active field of investigation during the contemporary period.

### Frail kidney phenotype: what is it?

The importance of frailty in geriatric practice and research has sparked substantial interest in applying this concept to those with other illnesses, especially chronic kidney disease (CKD). The prevalence of frailty increases from 8% - 16% in those with early CKD to 30% - 80% in those with end-stage kidney disease (ESKD) under chronic dialysis [10]. A literature summary we previously published, and cohort studies revealed that the increased susceptibility to frailty development in CKD population could be explainable by intertwining risk factors such as sociodemographic vulnerability, concurrent morbidities, polypharmacy, and malnutrition/protein-energy wasting [11-13]. Meta-analyses repeatedly show that frailty in these renal patients correlate with 1.5- to 2-fold higher risk of mortality, hospitalization, and fall episodes [10,14], and the adverse influences also apply to those with CKD and prefrailty. Besides, frailty is also found to impair bone health, predisposing CKD individuals to cognitive decline, depression, and dialysis access failure, paving the way toward greater healthcare consumption and costs [15-17]. Therefore, the concept of frailty in patients with CKD, alternatively termed “frail renal phenotype”, “frail kidney phenotype”, or “frail dialysis phenotype” [18,19], gains increasing attention worldwide, especially in developed countries with an aging population. This conceptualization has also been used in classifying patient eligibility for renal supportive care [20], supporting its widespread clinical applicability.

## Extracellular microRNAs (miRNAs) as biomarkers in various diseases

MiRNAs are small, single-stranded, and non-coding RNAs, with a size near 20 to 25 nucleotides, transcribed from different regions of the host genome, mostly intronic and intergenic ones [21]. RNA polymerase II and III assist in the transcription of primary miRNAs, which are later trimmed to precursor miRNAs by Drosha and DiGeorge Syndrome Critical Region 8 (DGCR8), followed by Dicer processing to miRNA duplex for proper action. MiRNAs function through loading onto the RNA-induced silencing complex for mRNA degradation and translational suppression, and their low requirement for base-pairing complementarity supports the existence of a wide range of target genes. More than 2000 different species of miRNAs have been identified in human beings, and it has been estimated that nearly 60% of human protein-coding genes are potentially regulated by these small RNA species [22].

### MiRNAs: from pathophysiologic regulators to biomarkers

MiRNAs participate extensively in developmental processes and physiologic regulation of organism function, and their derangement or aberrated expression patterns are involved in multiple chronic diseases, from cardiovascular, neoplastic to kidney diseases [23]. Starting from the examination of miRNA expressions in diseased tissues relative to controls, miRNA dysregulation is further affirmed to exhibit functional implications based on findings from experimental models. For example, we previously evaluated expressions of two miRNAs in vascular tissues and vascular smooth muscle cells to discern whether they were involved in CKD-associated vasculopathy, and tested our findings using functional assays, disclosing that their overexpression in vascular cells suppressed vasculopathy severities [24,25]. A recent review also nicely summarizes the conventional miRNA research pipeline, with various modifications based on disease specificity and resource availability [26].

Although miRNAs mainly exert biologic functions intracellularly, they are also detectable in extracellular fluids either in protein-attached forms or in extracellular vesicles. Since the first report of miRNA residing in the extracellular space [27], there is growing interest in harnessing extracellular miRNAs as disease biomarkers, judging from their pathogenic importance. Indeed, extracellular miRNAs in exosomes, or vesicles approximately 50-100 nm in diameter, are detectable through densitometric separation, ultracentrifugation, electron microscopy, and/or specific proteins on their surfaces [28]. Their role in intercellular communication and paracrine actions cannot be overstated. Non-vesicular extracellular miRNAs may attach to RNA-binding proteins (Argonaute 2 or high-density lipoprotein) in biologic fluids and therefore stay resistant to RNase degradation, amenable to steady state detection [29]. It is then plausible that we can leverage on the stability of extracellular miRNAs in biologic fluids, through using them as biomarkers reflecting disease-specific machineries and severity estimation. Ample number of extracellular miRNAs have been detected in sera, plasma, urine, saliva, cerebrospinal fluid, saliva, etc. [30], especially upon tissue injuries or during stress condition, further lending support to their clinical utility. Existing literature offers many experiences in this approach; Kinet and colleagues concluded that extracellular miRNAs assisted significantly in the diagnosis and prognostication of cardiovascular disease, including myocardial infarction, heart failure, and coronary artery disease [31]. Specific circulating miRNAs not only help classify individuals with greater severity of vascular calcification but also assist in predicting longitudinal disease trajectories [24,32]. Others also indicate that miRNAs in the circulation or bile juices are potential biomarkers for different types of liver diseases and for assessing hepatic injuries [33].

## MiRNAs and frail kidney phenotype

### miRNAs in frailty

MiRNAs reportedly participate in the initiation and progression of frailty from a biologic perspective. The development of sarcopenia, or age-/disease-related loss of skeletal muscle and a frequent co-existing phenotype with frailty, is accompanied by a strikingly large list of dysregulated miRNAs, followed by muscle protein homeostasis disturbance, myocyte energy insufficiency, myofibril transformation, and fatty infiltration [34]. Several function-based classes of miRNAs, such as senescence-associated miRs, inflamma-miRs, and hypoxa-miRs, are dysregulated in neurons, glial cells, and astrocytes, leading to alterations in synaptic function and oxidative injuries in brain tissues, culminating in cognitive impairment [35]. Targets of miRNAs putatively contributing to sarcopenia and frailty include phosphoinositide 3-kinase/protein kinase B, transforming growth factor-beta and SMAD family members, and insulin-like growth factor 1 [36]. Another opinion pieces further classifies frailty-contributing miRNAs according to their pathological machineries, into inflamma-miRs, myomiRs, and mitomiRs [37]. Apart from participating in the pathogenesis of frailty, miRNAs are also known to be influenced by exercise and dietary status, suggesting that they can be markers of these environmental exposures. D’Souza and colleagues found that in older adults, resistance exercise consistently modulated skeletal muscle miR-16-5p expressions, whereas protein supplementation also altered muscular miRNA levels [38]. Another report disclosed that a chronic multicomponent exercise prescription affected serum miR-93-5p, miR-155-5p, and miR-495-3p levels [39], lending support to the possibility that miRNAs act as surrogates of the multi-dimensional effects resulting in frailty.

As miRNAs are frequently packaged into vesicles and released extracellularly as means of inter-cellular communications, extracellular miRNAs are also purported as potential markers for frailty identification. Following the call of the FRAILOMIC consortium, investigators started to examine the utility of miRNAs as frailty biomarkers in their omic-based approach [40]. Carini and colleagues profiled whole blood miRNAs from 41 subjects without or with frailty, and discovered that circulating miR-101-3p and miR-142-5p were lower in frail individuals than robust ones [41]. A subsequent systematic review revealed five reports addressing extracellular miRNAs as circulating markers for frailty, including miR-10a-3p, miR-92a-3p, miR-185-3p, miR-194-5p, miR-326, miR-532-5p, miR-576-5p, and miR-760 [42]. Similar targets and implicated pathways of miRNA are uncovered, such as insulin signaling, FoxO pathways, and mitochondrial protein cargos. The findings thus echo those from tissue-based miRNAs involved in frailty pathogenesis, supporting the clinical potentials of miRNAs as biomarkers. Another recent study disclosed that serum miR-103a-3p, miR-598-3p, and miR-130a-3p levels were differentially expressed between robust and frail individuals with myocardial infarction [43]. Others pinpointed serum miR-451a levels were increased significantly among frail older adults, serving as a potential frailty biomarker [44]. An experimental report further showed that extracellular vesicles from young mesenchymal stem cells could prevent frailty and restore epigenetic ages among older littermates through their cargo of miRNA cocktails [45].

### miRNAs in frail kidney phenotype

Pathogenesis of frail kidney phenotype in patients with CKD involves a plethora of dysregulated physiologic systems. An important contributor to this phenotype is the uremic toxins, including small, large, and protein-bound ones [46]. These toxins precipitate organ-specific detrimental effects, through inducing inflammation, resident cell senescence, and exhaustion of repairing mechanisms, constituting a vicious cycle toward ESKD [46,47]. MiRNAs also play a role in frail kidney phenotype pathogenesis, although dedicated reports are very few. Clinically, a single center study indicated that subcutaneous and peritoneal adipocyte miR-130b and miR-17-5p expressions correlated with the risk of frailty and 2-year mortality among patients with advanced CKD [48]. Contributors to frail kidney phenotype, especially uremic sarcopenia, are also influenced by miRNA actions. Researchers speculated that miRNAs might affect the severity of CKD-induced muscle wasting through regulating myogenesis and myostatin expressions and altering myoblast differentiation [49]. The putative muscle-kidney axis, a crosstalk between muscular atrophy and kidney fibrosis, is established based on the observed action of miR-23a/27a, whose administration effectively restored skeletal muscle mass and ameliorated kidney damage [50]. There are reports suggesting that miR-26a suppression was associated with muscle atrophy in CKD through increasing insulin resistance [51], and miR-29 inhibition could attenuate myogenesis and predispose animals to the development of uremic sarcopenia [52].

MiRNAs also influence the severity of frail kidney phenotype through altering the progression of uremic vasculopathy. The downregulation of miR-125b-5p and miR-378a-3p is found to create a tendency toward vascular medial mineralization and vascular stiffening amidst uremic status [24,25], leading to compromised tissue perfusion and potentially frailty. Several miRNAs also correlate with markers of CKD-mineral bone disorder (CKD-MBD) such as osteoprotegerin, fibroblast growth factoe-23, and fetuin-A among ESKD patients, underlying their relationship with renal osteodystrophy, osteoporosis, and other metabolic bone disorder [53].

**Figure 1.**
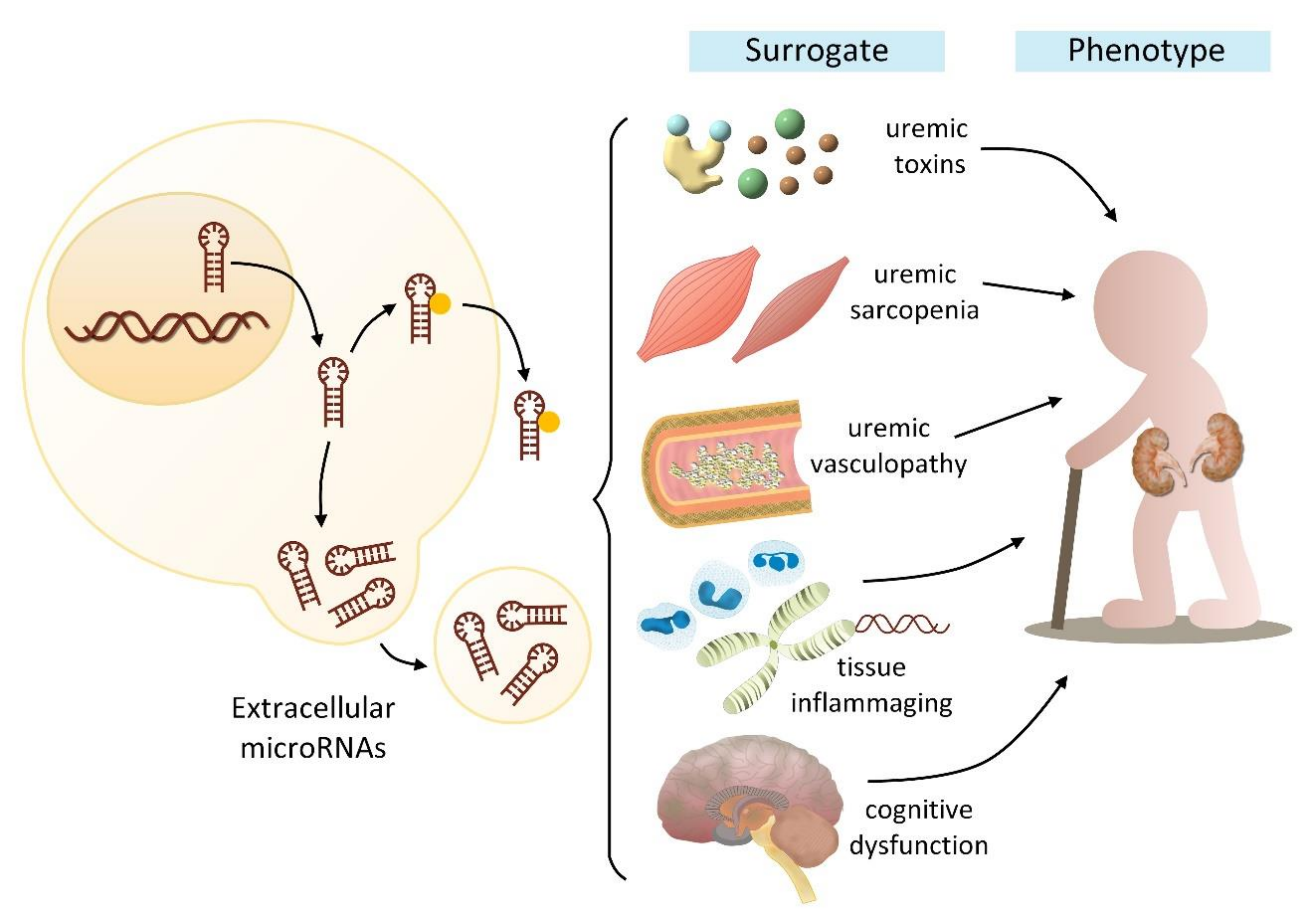
An illustrative diagram shows the utility of extracellular miRNAs as surrogates of frail kidney phenotype mediators and contributors.

## Extracellular miRNAs as biomarkers for frail kidney phenotype: status quo

Judging from the promising results obtained in miRNA-based frailty biomarker searches, it is theoretically attractive to test similar conceptual links in patients with CKD. However. studies very rarely, if identifiable, analyzed the association between extracellular miRNA levels and frail kidney phenotype. We propose a diagram to illustrate the potential utility of extracellular miRNAs as correlates and even predictors of frail kidney phenotype (Fig. 1); miRNAs in biological fluids may serve as surrogates for multiple predecessors of frail kidney phenotype, including the levels of specific uremic toxins *per se*, certain uremic organ complications, and molecular frail pathogenesis. Extrapolation of results from reports addressing miRNA biomarkers for vital contributors of frailty remains common and serves as a surrogate approach. For example, inflammaging is a culprit phenomenon responsible for frail kidney phenotype [47], and existing literature suggests that circulating miR-29b tightly correlated with inflammatory markers in patients with CKD [54]. Exosomal miRNAs, such as miR-16-5p, miR-17/92 cluster members, and miR-106-5p also exhibited a close relationship with vascular stiffness severity [55], a contributor to frailty in those with CKD [56]. Proatherogenic miRNAs such as miR-223 also exhibit an association with inflammation and vascular dysfunction in ESKD patients [57], predisposing them to a subtype of frailty termed vascular frailty [58]. Although none of the existing literature addresses the utility of extracellular microRNA as markers for uremic sarcopenia, there is an anecdotal report suggesting that serum extracellular vesicle miR-203a-3p independently correlated with skeletal muscle mass across different muscular use/disuse conditions [59]. Despite these encouraging findings, there remains a large research gap in directly affirming the connection between specific extracellular miRNAs and frail kidney phenotype. We are still in need of more evidence in this field.

**Table 1:** Precautions in conducting studies of extracellular miRNA as frail kidney phenotype biomarkers.

*Category*	*Description*
**Definition**	How frailty is assessed in the index population
**Functional/tissue relevance**	Specific miRNA(s) only offer limited contributions to frailty
**CKD severity**	1. The severity of CKD unanimously influences measured miRNA levels
	2. Dialysis treatment potentially influences miRNA levels
**Technical consideration**	1. miRNA terminology and annotation
	2. Biologic samples being tested
	3. Standardization/quantification methods

*CKD, chronic kidney disease*

## Precautions in miRNA applicability as biomarkers for frail kidney phenotype

Notwithstanding the above progress, important precautions require attention when conducting relevant researches (Table 1). These considerations can be divided into the following categories: frailty definition, functional/tissue specificity of miRNAs, the severity of CKD, and technical considerations. As explained in the first section, frailty can be assessed based on the phenotype or the index approach. The difference in the classification strategy poses substantial influence on assessment results, especially in those with CKD. A recent study showed that the agreement of frailty diagnosis between the phenotype and the index approach could be quite low (kappa = 0.09) in dialysis patients, although their mortality predictive efficacy was similar [60]. The sensitivity of various frailty-assessment tools also differs [61], and variations in results likely aggravate if the cutoff values for recognizing frailty change across studies. This phenomenon also arouses concerns regarding the feasibility of frailty assessment during routine practice [62]. Second, miRNA sources and actions are frequently tissue specific, implying that changes in their extracellular levels and the observed connections likely originate from differences in frailty pathogenic machineries or tissue functions. Single miRNA offers relatively insufficient information for frailty identification, whereas miRNA panels potentially perform better [63]. It is expected that combining miRNA pertaining to different pathologies and enriched tissues will exert better predictive effect. In addition, the prevalence and incidence of frail kidney phenotype tends to surge with worsening renal function, but circulating miRNA levels progressively decline [64]. This undoubtedly creates barriers to investigators, who seek to expand positive sample size but are faced with a shrinking pool of miRNAs. Hemodialysis procedures may influence serum miRNA levels as well, although not directly through filtration [65]. Finally, technical issues also interfere with the interpretability of extracellular miRNA levels. Annotated miRNAs exhibit distinct features for verification and biological effect ascertainment, and are frequently conserved across species [66], but recently introduced ones may not. Whether a reported miRNA truly exists and binds to Argonaute protein should be determined prior to being tested as a biomarker. In addition, the biologic fluids from which extracellular miRNAs are assayed count. Circulating miRNA levels in sera or plasma can be affected by the pre-processing time, since hemolysis causes a release of miRNAs from blood cells [67]. Choices of plasma or sera also influence the results; plasma miRNA levels can be higher than those from sera due to the associated subcellular components in the former [68]. If urinary miRNAs are being assayed, correction for urine concentration would be needed, using urine creatinine or total protein as a reference [69]. Experimental details regarding small RNA extraction (Trizol, or commercial kits), miRNA amplification and quantification (SYBR or TaqMan-based system, sources of primers), and internal normalization controls (spike-in ones, literature-based, profiling designated, model dependence, etc.) all play a role in influencing extracellular miRNA detection [26], and therefore should be carefully optimized, if possible.

## Conclusion and future perspectives

Extracellular miRNAs as biomarkers for disease identification, severity estimation, and prognosis prediction is a compelling trend. Since the discovery of their stability in biological fluids and the potential of long-term storage, extracellular miRNAs have become an important tool, progressing from one useful in research setting to one with clinical potential. There are at least ten extracellular miRNA species demonstrating predictive efficacy for frailty detection in older adults, each with its functional implication annotated. These successful experiences inspire subsequent work in uncovering candidate miRNA-based biomarkers for frail kidney phenotype, although only preliminary results are summarized in this perspective. However, we also outline important issues necessitating consideration prior to validating specific extracellular miRNA(s) for identifying those with frail kidney phenotype. With these in minds, we expect a fruitful era of miRNA diagnostics in the field of frailty in the near future.
